# Diabetes among Indigenous Orang Asli adults in Peninsular Malaysia: Prevalence and associated factors from Orang Asli Health Survey 2022

**DOI:** 10.1371/journal.pone.0352878

**Published:** 2026-07-02

**Authors:** Hasimah Ismail, Thamil Arasu Saminathan, Hamizatul Akmal Abd Hamid, Wan Kim Sui, Tania Gayle Robert Lourdes, Noor Ani Ahmad

**Affiliations:** 1 Institute for Medical Research, National Institutes of Health, Ministry of Health Malaysia, Shah Alam, Selangor, Malaysia; 2 Institute for Public Health, National Institutes of Health, Ministry of Health Malaysia, Shah Alam, Selangor, Malaysia; UKM: Universiti Kebangsaan Malaysia, MALAYSIA

## Abstract

**Background:**

Type 2 diabetes mellitus (T2DM) is a growing public health problem globally, and in Malaysia. Orang Asli, the Indigenous population in Peninsular Malaysia, often experience a disproportionate burden of diabetes, driven by social disadvantage, lifestyle transitions, and limited access to healthcare. Given that no nationwide diabetes assessment has been conducted among this under-represented group, this study therefore aimed to determine the prevalence of diabetes and its associated factors among Orang Asli adults in Peninsular Malaysia.

**Methods:**

The Orang Asli Health Survey (OAHS) 2022 was a nationwide cross-sectional survey with a complex sampling design conducted among Orang Asli adults living in non-institutional households across nine states of Peninsular Malaysia. A two-stage stratified sampling strategy was used, with locality (urban, fringe, remote) as the primary stratum and tribe (Senoi, Proto-Malay, Negrito) as the secondary stratum. Data were collected using structured interviewer-administered questionnaires and standardised clinical measurements. Diabetes was defined as self-reported physician-diagnosed diabetes or fasting blood glucose ≥7.0 mmol/L. Descriptive statistics, chi-square tests, and multiple logistic regression were performed using SPSS Version 20, accounting for the complex survey design. The adjusted odds ratios (AORs) and 95% confidence intervals (CIs) were reported.

**Results:**

A total of 9,206 Orang Asli adults participated in the diabetes module. The overall prevalence of diabetes was 16.1% (95% CI: 14.3–17.9). Diabetes prevalence was higher among the females (16.8%), those aged ≥60 years (28.1%), urban residents (21.1%), Proto-Malay (18.8%) and Senoi (14.0%) tribes, participants with no formal or incomplete primary education (19.1%), those with monthly household income ≥RM2000 (21.2%), obese individuals (21.1%), respondents with hypertension (24.2%) and those with high total cholesterol (21.7%). In multivariable analysis, diabetes was significantly associated with age 40–59 years (AOR 1.64; 95% CI: 1.35–1.99) and ≥60 years (AOR 2.14; 95% CI: 1.40–3.28), urban (AOR 1.73; 95% CI: 1.07–2.79) and fringe (AOR 1.63; 95% CI: 1.13–2.35) localities, Senoi (AOR 1.70; 95% CI: 1.08–2.68) and Proto-Malay (AOR 2.41; 95% CI: 1.50–3.86) tribes, incomplete primary education (AOR 1.28; 95% CI: 1.02–1.60), household income ≥RM2000 (AOR 1.47; 95% CI: 1.12–1.93), hypertension (AOR 1.42; 95% CI: 1.03–1.95) and hypercholesterolemia (AOR 2.17; 95% CI: 1.70–2.76).

**Conclusions:**

Diabetes is common among Orang Asli adults in Peninsular Malaysia. Older age, urban and fringe residence, specific tribal groups, lower education, higher income, hypertension, and hypercholesterolaemia were associated with diabetes. These findings highlight the need for culturally appropriate diabetes prevention, screening, and management strategies tailored to Orang Asli communities, particularly for the elder subgroup and those living in urbanised settings.

## Introduction

Diabetes mellitus is a primary global public health concern. The International Diabetes Federation estimates that approximately 537 million adults were living with diabetes in 2021, with the burden rising most rapidly in low- and middle-income countries [1]. Malaysia mirrors this trend. According to the National Health and Morbidity Survey (NHMS) 2023, about one in six Malaysian adults has diabetes, reflecting a persistently high disease burden nationwide [2]. A recent systematic review and meta-analysis estimated that the pooled prevalence of type 2 diabetes mellitus (T2DM) in Malaysia was approximately 14%, with prediabetes affecting more than 10% of the adult population [3]. These findings underscore the need for enhanced national strategies to prevent, screen, and effectively manage the long-term disease.

Orang Asli, the Indigenous peoples of Peninsular Malaysia, represent a small but highly marginalised population. Many communities live in remote or fringe settlements and continue to experience socioeconomic disadvantages, including higher poverty levels, food insecurity, and limited access to healthcare and education [4]. Historically, research among the Orang Asli has focused on infectious diseases, undernutrition, and maternal–child health. However, recent evidence demonstrates a growing burden of non-communicable diseases (NCDs) within this population, including hypertension, dyslipidaemia, and diabetes, likely reflecting rapid social, environmental, and dietary transitions [5–7].

Published evidence on diabetes among the Orang Asli is limited and fragmented. Most existing studies have been small-scale and confined to specific villages, resettlement schemes, or isolated sub-tribes. Reported diabetes prevalence has varied widely, from less than 5% in groups that maintain more traditional, physically active lifestyles to more than 20% in semi-urban or resettled populations experiencing greater exposure to processed foods and sedentary living [6,8–10]. A recent systematic review of health conditions among the Orang Asli highlighted a rise in metabolic syndrome and impaired fasting glucose, noting that cardiometabolic risk profiles in some sub-groups have exceeded those of the general Malaysian population [6]. These findings collectively suggested that the Orang Asli may be undergoing an epidemiological transition characterised by increasing susceptibility to lifestyle-related chronic diseases.

Several factors may contribute to the rising diabetes burden in this population. Dietary patterns have shifted from traditional, forest-based foods toward greater consumption of processed, energy-dense products high in sugar and fat [11]. Changes in livelihood, moving from hunting, gathering, and shifting cultivation to wage-based or sedentary work, have led to a reduction in physical activity levels. Broader structural issues, including persistent poverty, poor health literacy, geographical isolation, and limited health service availability, may delay diagnosis and continuity of care. Global literature indicates that Indigenous populations commonly experience a combination of biological vulnerability, social disadvantage, and environmental stressors that increase both the risk and severity of diabetes [12]. Malaysian national data also show that older age, obesity, hypertension, dyslipidaemia, and residence in more urbanised areas are key determinants of diabetes [2,13]. The interaction of these factors within Orang Asli communities, which differ substantially in culture, settlement patterns, and socioeconomic conditions, remains poorly understood.

Importantly, there has been no nationally representative study of diabetes among Orang Asli adults. Existing research lacks the scale and diversity necessary to account for the three major tribal groupings, Senoi, Proto-Malay, and Negrito, and the varying localities (urban, fringe, and remote). The Orang Asli Health Survey (OAHS) 2022 was designed to fill this gap and represents the first nationwide effort to document the health status, risk factors, and NCD patterns across all Orang Asli sub-groups in Peninsular Malaysia [14].

Using data from OAHS 2022, this study aimed to estimate the prevalence of diabetes among Orang Asli adults and to identify sociodemographic and clinical parameters that are associated with diabetes in this population. We hypothesised that, similar to patterns observed in the general Malaysian population, older age, metabolic risk factors such as hypertension and hypercholesterolaemia, and residence in more urbanised areas would increase the odds of diabetes. The findings will hence inform culturally appropriate prevention, early detection, and clinical management strategies tailored to the specific needs of Orang Asli communities.

## Methods

### Study design and population

The Orang Asli Health Survey (OAHS) 2022 was a nationwide, cross-sectional study with a complex sampling design, conducted by the Institute for Public Health (IPH) of the Ministry of Health Malaysia. The survey targeted all Orang Asli residents living in non-institutional living quarters for at least two weeks. Individuals residing in institutions such as hospitals, hostels, hotels, or nursing homes were excluded from the study. The sampling frame was derived from the Department of Orang Asli Development (JAKOA) database, which included Orang Asli communities from nine states in Peninsular Malaysia: Selangor, Perak, Kedah, Melaka, Negeri Sembilan, Johor, Kelantan, Terengganu, and Pahang. Altogether, 853 Orang Asli villages were identified and categorised into three geographical strata: urban, fringe, and remote. Further information regarding OAHS sampling procedures, survey logistics, and operational implementation is available in the OAHS 2022 Technical Report published by IPH in 2024 [14].

### Sampling strategy

A two-stage stratified sampling approach was employed. In the first stage, the villages were selected proportionately to size within each of the three locality strata. In the second stage, the major Orang Asli tribal groups, Senoi, Proto-Malay, and Negrito, served as secondary strata to ensure representation of each main sub-population. All eligible adults aged 18 years and above who were usual residents of the selected households were invited to participate in the survey.

### Data collection procedures

Data collection for OAHS 2022 was conducted from 20th July 2022 and 13th September 2022. Data collection was conducted by trained interviewers using structured, interviewer-administered questionnaires. Interviews were carried out face-to-face using mobile data capture devices linked to a secure OAHS server. Completed questionnaires were synchronised with the central database whenever internet access was available. Quality assurance procedures included real-time verification of respondent identification numbers, automated range and consistency checks, and manual review of outliers and entries that were questionable. Clinical assessments were performed by trained research assistants and qualified nurses following standardised procedures. Written informed consent was obtained from all respondents before administering any questionnaires or clinical measurements, before the commencement of the data collection procedure.

### Clinical measurements and laboratory procedures

Fasting capillary blood glucose was measured using the CardioChek® PA Analyzer. This point-of-care device has been independently validated and demonstrated acceptable accuracy for population screening when compared with laboratory reference methods [15]. Diabetes was defined according to the World Health Organization diagnostic cut-off of fasting plasma glucose ≥7.0 mmol/L [16]. Respondents were classified as having diabetes if they self-reported a prior diagnosis by a doctor or assistant medical officer, or if their fasting capillary glucose result met the WHO threshold.

Anthropometric measurements were performed using calibrated equipment. Body weight was measured to the nearest 0.1 kg using a SECA 813 digital weighing scale, and height was measured to the nearest 0.1 cm using a SECA 213 stadiometer. Body mass index (BMI) was calculated as weight in kilograms divided by height in metres squared. Obesity was defined as a BMI of 30.0 kg/m^2^ or higher, according to the World Health Organization’s adult cut-off [17]. Blood pressure was measured using the Omron HEM-907 device. Three measurements were taken at one-minute intervals, and the average of the second and third readings was used for analysis. Respondents were classified as hypertensive if their mean systolic blood pressure was ≥ 140 mmHg, diastolic blood pressure was ≥ 90 mmHg, or if they self-reported a previous diagnosis of hypertension. Total cholesterol was assessed using the CardioChek Analyzer via finger-prick sampling. Hypercholesterolemia was defined as a total cholesterol level of≥5.2 mmol/L. All clinical values obtained by the nurses were first recorded on clinical assessment forms and subsequently entered into the digital data collection system by research assistants.

### Independent variables

Sociodemographic characteristics included sex, age group (18–39 years, 40–59 years, and 60 years and above), locality (urban, fringe, remote), tribal group (Senoi, Proto-Malay, Negrito), education level (never attended school or no formal education, not completed primary school, completed primary school, completed Form 3, and at least completed Form 5), marital status (unmarried or married/living with a partner), occupational status (employed, housewife or homemaker, student or not working), and monthly household income (<RM500, RM500–999, RM1000–1999, and ≥RM2000). Health-related variables included obesity, hypertension, hypercholesterolaemia, current smoking status, and current alcohol consumption status. Current smoking was defined as self-reported current use of tobacco products. Current alcohol consumption was defined as self-reported current intake of alcoholic beverages.

### Statistical analysis

This manuscript is a secondary analysis of anonymised OAHS 2022 data. The research team accessed the anonymised dataset for analysis in January 2025. No personally identifiable information was available to authors at any stage. All data were entered, cleaned, and analysed using SPSS Version 20 (SPSS Inc., Chicago, IL, USA). Descriptive analyses were used to summarise respondents’ characteristics and estimate the prevalence of diabetes by sociodemographic and clinical variables. Pearson’s chi-square test was used to examine the association between categorical variables and diabetes status. Crude odds ratios and their corresponding 95% confidence intervals were calculated in bivariable analyses.

Multiple logistic regression was performed to identify factors independently associated with diabetes while controlling for potential confounders. Variables were selected based on statistical significance in bivariable analyses and changes in the −2 log-likelihood value. The final model included sex, age group, locality, tribal group, education level, marital status, occupational status, household income, obesity, hypertension, and cholesterol status. Adjusted odds ratios and 95% confidence intervals were reported, with a p-value less than 0.05 considered statistically significant.

### Ethical considerations

This study involved secondary analysis of data from the Orang Asli Health Survey (OAHS) 2022. The original OAHS protocol was approved by the Medical Research and Ethics Committee (MREC), Ministry of Health Malaysia (NMRR-19-3108-50999). Approval to access Orang Asli communities and sampling frames was granted by the Department of Orang Asli Development (JAKOA), Ministry of Rural and Regional Development. Written informed consent was obtained from all participants during the OAHS 2022 data collection. No additional data were collected for this manuscript. All datasets provided for analysis were fully anonymised by the Institute for Public Health (IPH), and the authors did not have access to identifiable data. Additional information regarding the ethical, cultural, and scientific considerations specific to inclusivity in global research is included in the Supporting Information (S1 Checklist).

## Results

### Socio-demographic characteristics

A total of 9,206 Orang Asli adults participated in the OAHS 2022 diabetes module. Women constituted a larger proportion of respondents (56.2%) compared with men (43.8%). The age distribution showed that 62.0% of respondents were between 18 and 39 years old, 28.8% were aged 40–59 years, and 9.2% were aged 60 years or older. Nearly half of the respondents (46.9%) resided in fringe localities, followed by remote areas (44.3%), while only 8.8% lived in urban areas.

In terms of tribal distribution, Senoi comprised a major proportion (41.8%), followed by Proto-Malay (35.2%) and Negrito (23.0%). By educational level, 25.4% had never attended school or had no formal education, 16.9% had not completed primary school, 25.5% had completed primary school, 16.6% had completed Form 3, and 15.6% had at least completed Form 5. Most respondents were married or living with a partner (80.1%). The employment status showed that 55.8% were employed, 30.8% were housewives or homemakers, and 13.4% were students or not in the workforce. Household income varied, with 42.5% reporting an income of less than RM500 per month, 21.2% earning between RM500 and RM999, 29.0% earning between RM1,000 and RM1,999, and 7.3% earning RM2,000 or more per month (Table 1).

**Table 1:** Socio-demographic characteristics of the respondents (N = 9206).

Socio-demographic characteristics	Count (n)	Percentage (%)
**Gender**		
Male	4033	43.8
Female	5173	56.2
**Age group**		
18-39	5712	62.0
40-59	2652	28.8
60 & above	842	9.
**Locality/Strata**		
Urban	806	8.8
Fringe	4319	46.9
Remote	4081	44.3
**Tribe group**		
Senoi	3847	41.8
Proto Malay	3244	35.2
Negrito	2115	23.0
**Education level**		
Never went to school/No Formal Education	2324	25.4
Not completed primary school	1545	16.9
Completed primary school	2334	25.5
Completed secondary 3	1138	12.4
At least completed secondary 5	1818	19.8
**Marital status**		
Unmarried	1677	19.9
Married/Live with a partner	6752	80.1
**Occupation status**		
Employed	5126	55.8
Housewife or Homemaker	2824	30.8
Student or not working	1233	13.4
**Monthly household income**		
Less than RM500	3909	42.5
RM500 – RM999	1951	21.2
RM1000–1999	2662	29.0
RM 2000 or more	667	7.3

### Prevalence of diabetes

The overall prevalence of diabetes among Orang Asli adults was 16.1% (95% CI: 14.33–17.94). Diabetes prevalence increased substantially with age: 10.9% among adults aged 18–39 years, 22.0% among those aged 40–59 years, and 28.1% among those aged 60 years or older. Prevalence differed across localities, with urban residents showing the highest prevalence (21.1%), followed by fringe residents (17.5%) and remote residents (11.0%).

Considerable variation was also observed across the tribal groups. Proto-Malay adults had the highest prevalence (18.8%), followed by Senoi (14.0%) and Negrito (6.4%). Diabetes prevalence was elevated among respondents with lower education levels: 19.1% among those who had never attended school or had no formal education, and 19.1% among those who had not completed primary school. By contrast, prevalence was lower among adults who had completed primary school (15.6%), completed Form 3 (12.2%), or at least completed Form 5 (approximately 13–15%).

Household income showed a positive gradient with diabetes. Adults in households earning RM2000 or more had the highest prevalence (21.2%), compared with 14.4–17.0% among those in lower income groups. Prevalence among obese respondents was 21.1% compared with 15.0% among non-obese respondents.

Diabetes was also more common among adults with hypertension (24.2%) than among those who were normotensive (12.3%). Similarly, adults with high total cholesterol prevalence of 21.7%, compared with 11.3% among those with normal cholesterol levels (Table 2).

**Table 2:** Prevalence of diabetes by socio-demographic characteristics (N = 9206).

**Demographic Characteristics**	**Count (n)**	**Estimated Population**	**Prevalence (%)**	**95 CI %**	
				**Lower**	**Upper**
**Overall**	1226	17359	16.1	14.33	17.94
**Gender**					
Male	486	7009	15.0	12.96	17.35
Female	740	10350	16.8	15.05	18.77
**Age group**					
18-39	491	6951	10.9	8.83	13.28
40-59	517	7224	22.0	20.13	24.07
60 & above	218	3184	28.1	23.90	32.80
**Residence/ Strata**					
Urban	170	251	21.1	18.57	23.85
Fringe	641	14386	17.5	15.68	19.52
Remote	415	2722	11.0	7.84	15.12
**Tribe group**					
Senoi	516	7941	14.0	12.15	16.12
Proto Malay	572	9277	18.8	16.62	21.23
Negrito	138	141	6.4	4.41	9.24
**Education level**					
Never went to school/No Formal Education	378	5404	19.1	16.35	22.24
Not completed primary school	221	3428	19.1	15.89	22.89
Completed primary school	302	4099	15.6	13.05	18.64
Completed Form 3	128	1553	12.6	10.19	15.51
At least completed Form 5	192	2768	12.2	9.06	16.17
**Marital status**					
Unmarried	178	2467	12.0	9.70	14.70
Married/Live with a partner	911	12732	16.5	14.71	18.44
**Occupation status**					
Employed	633	9298	14.6	12.71	16.83
Housewife or Homemaker	387	5220	16.9	14.62	19.43
Student or not working	200	2732	20.6	17.16	24.63
**Monthly household income**					
Less than RM500	491	7294	15.4	13.73	17.27
RM500 – RM999	241	3764	16.0	13.52	18.81
RM1000–1999	338	4507	15.5	12.33	19.29
RM 2000 or more	150	1640	21.2	17.65	25.23
**Obesity**					
Non-Obese	833	11941	15.0	13.07	17.13
Obese	353	4825	21.1	16.96	26.03
**Hypertension status**					
Normotensive	616	9119	12.3	9.76	15.44
Hypertensive	610	8240	24.2	21.74	26.81
**Cholesterol status**					
Normal cholesterol	467	6598	11.3	9.96	12.73
Hypercholesterolaemia	759	10761	21.7	18.89	24.80
Current smoker					
Yes	327	4927	14.4	12.15	17.05
No	843	11747	16.6	14.74	18.7
Current alcohol consumption					
Yes	98	1832	16.3	11.57	22.36
No	1128	15527	16	14.35	17.87

### Factors associated with diabetes

Multiple logistic regression identified several independent determinants of diabetes among Orang Asli adults. Age remained a strong predictor: compared with adults aged 18–39 years, those aged 40–59 years had significantly higher odds of diabetes (AOR 1.64; 95% CI: 1.35–1.99), and those aged 60 years or older had more than twice the odds (AOR 2.14; 95% CI: 1.40–3.28).

Locality was also significantly associated with diabetes. Compared with adults living in remote areas, the odds of diabetes were higher among those in fringe localities (AOR, 1.63; 95% CI, 1.13–2.35) and highest among those in urban areas (AOR, 1.73; 95% CI, 1.07–2.79).

Tribal differences persisted even after adjustment. Senoi adults had 1.70 times higher odds of diabetes (95% CI: 1.08–2.68), while Proto-Malay adults had 2.41 times higher odds (95% CI: 1.50–3.86), compared with Negrito adults.

Education level showed a significant association. Adults who had not completed primary school had higher odds of diabetes (AOR 1.28; 95% CI: 1.02–1.60) than those who had at least completed Form 5. Other education categories were not significantly associated with diabetes after adjustment for confounding factors.

Household income of RM2000 or more remained significantly associated with diabetes (AOR 1.47; 95% CI: 1.12–1.93), compared with income below RM500.

Hypertension and hypercholesterolaemia also showed strong associations. Hypertensive adults had higher odds of diabetes (AOR 1.42; 95% CI: 1.03–1.95). Adults with high total cholesterol had more than double the odds of diabetes (AOR 2.17; 95% CI: 1.70–2.76). Although obesity was associated with diabetes in crude analysis, the association did not remain statistically significant in the adjusted model (AOR 1.31; 95% CI: 0.91–1.88). Smoking status and current alcohol consumption were not significantly associated with diabetes in both crude and adjusted analyses (Table 3).

**Table 3:** Factors associated with diabetes.

Risk Factors	Crude OR				Adjusted OR			
	Exp (B)	95 CI %		p-value	Exp (B)	95 CI %		p-value
		Lower	Upper			Lower	Upper	
**Gender**								
Male	0.87	0.76	1.01	0.060	1.04	0.86	1.27	0.677
Female	1.00				1.00			
**Age group**								
18-39	1.00				1.00			
40-59	2.32	1.79	3.01	<0.001	1.62	1.32	1.99	0.000
60 & above	3.22	2.60	3.97	<0.001	2.12	1.35	3.32	0.001
**Residence/ Strata**								
Urban	2.17	1.45	3.25	<0.001	1.79	1.12	2.86	0.016
Fringe	1.73	1.17	2.56	0.007	1.67	1.17	2.39	0.005
Remote	1.00				1.00			
**Tribe group**								
Senoi	2.38	1.55	3.65	<0.001	1.68	1.06	2.67	0.029
Proto Malay	3.38	2.21	5.16	<0.001	2.35	1.47	3.75	0.001
Negrito	1.00				1.00			
**Education level**								
Never went to school/ No Formal Education	1.71	1.14	2.55	0.010	1.28	0.97	1.69	0.083
Not completed primary school	1.71	1.23	2.38	0.002	1.32	1.05	1.66	0.017
Completed primary school	1.34	0.91	1.96	0.131	1.32	1.02	1.70	0.037
Completed Form 3	1.04	0.74	1.47	0.814	0.93	0.70	1.24	0.630
Completed form 5	1.00				1.00			
**Marital status**								
Unmarried	1.00				1.00			
Married/Live with a partner	1.45	1.19	1.77	<0.001	1.07	0.84	1.37	0.584
**Occupation status**								
Employed	0.66	0.54	0.81	<0.001	0.89	0.61	1.30	0.538
Housewife or Homemaker	0.78	0.61	1.00	0.047	1.10	0.70	1.72	0.668
Student or not working	1.00				1.00			
**Monthly household income**								
Less than RM500	1.00				1.00			
RM500 – RM999	1.04	0.87	1.26	0.647	1.00	0.77	1.30	0.981
RM1000–1999	1.01	0.76	1.32	0.968	1.07	0.79	1.44	0.662
RM 2000 or more	1.48	1.12	1.95	0.007	1.44	1.11	1.86	0.007
**Obesity**								
Non-Obese	1.00				1.00			
Obese	1.52	1.12	2.06	0.007	1.33	0.91	1.92	0.135
**Hypertension status**								
Hypertensive	2.27	1.68	3.07	<0.001	1.41	0.99	2.01	0.056
Normotensive	1.00				1.00			
**Cholesterol status**								
High cholesterol	2.18	1.79	2.66	<0.001	2.12	1.66	2.71	0.000
Normal cholesterol	1.00				1.00			
Current smoker								
Yes	0.85	0.72	0.99	0.035	0.85	0.73	1.00	0.055
No	1.00				1.00			
Current drinker								
Yes	1.02	0.69	1.49	0.930	1.22	0.84	1.78	0.286
No	1.00				1.00			

Significant difference at *α =* 0.05.

Analysis was done using complex sample logistic regression analysis. The classification table (84.2%) was used to check model fitness.

## Discussion

This study has provided the first nationally representative estimate of diabetes prevalence among Orang Asli adults in Peninsular Malaysia. The overall prevalence of 16.1% is comparable to, and slightly higher than, the prevalence observed in the general Malaysian adult population in NHMS 2023 [2]. This finding is important because it indicates that despite being a smaller and historically underserved population, the Orang Asli experience a diabetes burden that is at least equivalent to that of the broader population, and likely rising. The results add substantial new evidence to a literature that previously relied on small, localised, and often non-representative studies.

Earlier studies among the Orang Asli communities reported a much lower prevalence of diabetes. In the early 1990s, Osman et al. reported that only 1.3% of Orang Asli adults had diabetes [18]. In 2010, Wan Nazaimoon and Suraiami documented 8.4% prevalence of diabetes and 16.8% of impaired fasting glucose among Orang Asli women [19]. Subsequent village-based studies reported prevalence estimates ranging from 4.6% to 16.8%, depending on the locality and tribal group [20,21]. A more recent systematic review by Mahmud et al. highlighted the increasing prevalence of metabolic derangement, including impaired fasting glucose and metabolic syndrome, across various Orang Asli sub-groups [6]. The present findings confirmed that diabetes has become a significant health concern among Orang Asli adults nationwide.

Age emerged as one of the strongest predictors of diabetes. Adults aged 40–59 years had significantly higher odds of diabetes compared to younger adults; and those aged 60 years and above had more than twice the odds. Age-associated deterioration in pancreatic β-cell function, increased insulin resistance, changes in body composition, and reduced physical activity are well-recognised contributors to rising diabetes risk in older adults [15,22]. These findings are consistent with ample national and international evidence that demonstrates the strong influence of age on diabetes prevalence [2,23–25,26].

Locality also played a notable role. The prevalence of diabetes was higher in urban and suburban areas than in rural or remote localities. This pattern mirrors global trends showing that Indigenous groups living closer to urban environments often experience greater exposure to processed foods, higher-calorie diets, and sedentary lifestyles [12,27]. Orang Asli communities in fringe and urban settings may also have greater accessibility to store-bought food and sweetened beverages, which have been linked to a higher risk of diabetes [11,20]. In contrast, Orang Asli living in remote settlements may retain more traditional dietary and activity patterns that confer some protection.

Significant differences were also observed across tribal groups, with Proto-Malay and Senoi adults showing higher odds of diabetes compared with Negrito adults. These findings align with earlier studies suggesting that Orang Asli sub-groups differ in their degree of contact with mainstream society, dietary patterns, levels of acculturation, and socioeconomic conditions [4,6,20,21]. Proto-Malay and Senoi communities tend to live closer to populated areas and roads compared to many Negrito settlements, potentially increasing their exposure to dietary transition and sedentary behaviour.

The education level exhibited a transparent gradient in the adjusted analysis. Adults who had not completed primary school had significantly higher odds of diabetes compared with those who had at least completed Form 5. Lower educational attainment is often linked to limited health literacy, reduced awareness of NCD risk factors, and delayed uptake of health services [23,24,28]. Interestingly, a higher household income (≥RM2000) was associated with an elevated risk of diabetes. Similar patterns have been observed in other Indigenous and lower-income populations undergoing socioeconomic transition, whereby rising household income increases accessibility to processed foods before improvements in health literacy and healthcare utilisation occur [24,25,29,30]. This could reflect an early stage of the “nutrition transition,” characterised by increased consumption of commercial foods and reduced reliance on traditional diets [11].

Hypertension and hypercholesterolaemia were strongly and independently associated with diabetes. This clustering of cardiometabolic risk factors is widely documented in global populations [31,22,30,32–34]. Hypercholesterolaemia in particular demonstrated a robust association, with affected individuals showing more than double the odds of diabetes. These findings are consistent with evidence that dysregulated lipid metabolism contributes to insulin resistance and early β-cell dysfunction [22,33]. The co-occurrence of diabetes, hypertension, and dyslipidaemia is known to accelerate the development of atherosclerotic cardiovascular disease and increase the risk of chronic kidney disease and premature mortality [32–34]. These results underscore the importance of integrated cardiometabolic screening and management in the Orang Asli communities.

Obesity, although associated with diabetes in crude analysis, did not retain statistical significance in the multivariable model. Several explanations are possible. BMI may be a less accurate measure of adiposity among Indigenous populations with differing body composition or fat distribution patterns. Central obesity as a more relevant metabolic marker was not assessed in the present study. It is also possible that residual confounding from diet, physical activity, or genetic factors influenced this relationship [12,30,22]. Similar findings have been reported in some Indigenous groups where diabetes occurs at lower BMI thresholds than in non-Indigenous populations [12,18].

Lifestyle-related behavioural variables such as diet and physical activity were not available in the OAHS 2022 dataset and therefore could not be evaluated in this analysis. Smoking and current alcohol consumption were assessed but were not significantly associated with diabetes after adjustment. The absence of detailed dietary and physical activity information limits the interpretation of behavioural pathways contributing to diabetes risk among Orang Asli communities, particularly in the context of rapid socioeconomic and nutritional transition. Future nationally representative studies should incorporate comprehensive lifestyle assessments to better characterise modifiable determinants of diabetes in this population.

This study has several strengths. It is the first to use a nationally representative sample of Orang Asli adults, encompassing all major tribal groups and locality strata. Standardised procedures were applied for clinical assessments, including fasting glucose, blood pressure, and cholesterol measurements, enhancing data reliability. The large sample size enables more precise estimates of diabetes prevalence and its associated factors across diverse subgroups.

However, several limitations should be acknowledged. The cross-sectional design precludes causal inference, and associations should be interpreted cautiously. Some variables relied on self-reports, which may introduce recall bias or underreporting. Fasting capillary glucose, although validated and practical for field settings, may underestimate diabetes compared to venous plasma glucose or HbA1c. Although smoking and current alcohol consumption variables were available and assessed, detailed dietary intake and physical activity variables were not collected in OAHS 2022. Therefore, important behavioural pathways associated with diabetes could not be fully evaluated.

Despite these limitations, the findings clearly show that diabetes is a significant and growing health issue among the Orang Asli. The identified associations, particularly those involving age, locality, tribe, education, income, hypertension, and cholesterol, highlight specific groups that may benefit most from targeted interventions. Improved access to health information, regular NCD screening, culturally tailored education programmes, and strengthening of linkage to primary health care could help reduce the burden of diabetes in this underserved population.

## Implications

The findings of this nationally representative study highlighted several important implications for public health planning, policy formulation, and clinical service delivery for Orang Asli communities. The high prevalence of diabetes, together with the sociodemographic and metabolic correlates identified, suggested that the Orang Asli are experiencing a rapid epidemiological transition similar to other Indigenous populations globally. Addressing this growing burden will require coordinated health system interventions, intersectoral collaboration, and community engagement.

The strong association observed between diabetes and other cardiovascular risk factors, such as hypertension and hypercholesterolaemia, underscores the importance of integrated NCD management rather than condition-specific programmes. International evidence shows that coordinated approaches focusing on early detection, risk stratification, and comprehensive management yield better outcomes than isolated disease-specific strategies [22,32–34]. Within Orang Asli settlements, especially in fringe and urban areas where metabolic risk is highest, primary care teams should adopt combined screening for diabetes, hypertension, and lipid disorders. Outreach services delivered through mobile clinics or scheduled community visits may increase uptake, particularly among households with lower educational levels or those residing in more remote locations.

The study highlights apparent differences in diabetes risk by locality and tribal group. Orang Asli communities in urban and fringe areas, who experience greater exposure to processed foods and sedentary behaviour, should be prioritised for tailored health promotion activities. Programmes that incorporate culturally meaningful messages, integrate local language materials, and involve community leaders have shown greater acceptability and effectiveness among Indigenous populations [12,27]. In Malaysia, strengthening partnerships between the Ministry of Health, the Department of Orang Asli Development (JAKOA), and community representatives could help build trust and increase participation in screening and treatment programmes.

The association between higher household income and increased diabetes risk suggests that Orang Asli communities may be in an early phase of the nutrition transition, characterised by increased consumption of commercial, energy-dense foods. Studies across low- and middle-income countries have shown that rising income often leads to substitution away from traditional diets toward processed foods before health literacy and preventive care improve [24,30]. Policies that promote healthier food environments, including subsidies for healthier staples, limiting the availability of sugary beverages, and supporting community food programmes, may mitigate this trend. National findings also suggest that Orang Asli communities may benefit from targeted nutrition interventions aimed at reducing refined carbohydrate intake and encouraging consumption of traditional, nutrient-rich foods.

Improving health literacy is another critical area. Lower education levels were associated with higher odds of diabetes, consistent with evidence from multiple settings showing that education influences awareness of NCD symptoms, screening uptake, and treatment adherence [23,24,28]. Educational initiatives targeting individuals with limited formal schooling should use clear, simple communication and interactive methods. Community health promoters, including trained Orang Asli health volunteers, may bridge the gaps in understanding and promote consistent follow-up.

The continuity of care is an essential element in the healthcare system. Many Orang Asli communities face geographic and financial barriers in accessing clinics, leading to delayed treatment and suboptimal glycaemic control. Strengthening referral pathways, offering transport allowances, or integrating diabetes care into existing community programmes may improve outcomes. Experiences from Indigenous health initiatives in Australia, Canada, and Latin America have demonstrated that culturally adapted chronic disease management programmes can lead to significant improvements in glycaemic control, medication adherence, and cardiovascular risk profiles [12,22,32].

Lastly, there is a pressing need for longitudinal research to monitor the long-term progression of diabetes and cardiometabolic risk among the Orang Asli. Prospective studies would provide insight into causal pathways, especially regarding diet, physical activity, psychosocial stress, and genetic predisposition. Qualitative research exploring perceptions of diabetes, traditional beliefs, and barriers to healthcare utilisation would enrich understanding and support the development of more culturally appropriate interventions.

Overall, the findings suggest the need for an integrated, culturally sensitive, and community-driven approach to diabetes prevention and management among the Orang Asli population. Without targeted action, diabetes and its complications are likely to increase, widening existing health disparities.

## Conclusion

This nationally representative study provides the most comprehensive assessment to date of diabetes prevalence and associated factors among Orang Asli adults in Peninsular Malaysia. The findings demonstrate that diabetes is a significant and growing health issue within this population, with a prevalence of 16.1%, comparable to and slightly higher than that of the general Malaysian population. Older age, residence in urban and fringe localities, belonging to the Senoi or Proto-Malay tribes, lower educational attainment, higher household income, hypertension, and hypercholesterolaemia were independently associated with increased odds of diabetes. These patterns suggest that the Orang Asli are experiencing a rapid epidemiological transition influenced by both socioeconomic and lifestyle changes.

The results underscore the importance of developing tailored and culturally responsive strategies for diabetes prevention, early detection, and integrated management in the Orang Asli communities. Strengthening outreach services, improving access to primary care, and promoting health literacy, particularly among those with limited formal education, is crucial for addressing the diabetes burden. Without targeted intervention, the increasing prevalence of diabetes and its related complications may exacerbate existing health disparities and place additional strain on healthcare providers serving Indigenous populations.

This study establishes essential evidence to guide policy action and community-level interventions. Continuous monitoring and expansion of future research, including longitudinal and qualitative analyses, will be vital to understanding the evolving health challenges faced by the Orang Asli and to ensure that interventions remain effective, culturally aligned, and sustainable.

## Supporting information

S1 Checklist Inclusivity in global research questionnaire. (DOCX)

## References

[1] Magliano DJ, Boyko EJ. IDF Diabetes Atlas. 10th ed. IDF Diabetes Atlas 10th edition scientific committee. Brussels: International Diabetes Federation. 2021.

[2] Institute for Public Health (IKU). National Health and Morbidity Survey (NHMS) 2023: Non-Communicable Diseases and Healthcare Demand. Selangor: Ministry of Health Malaysia. 2024.

[3] Akhtar S, Nasir JA, Ali A, Asghar M, Majeed R, Sarwar A. Prevalence of type-2 diabetes and prediabetes in Malaysia: A systematic review and meta-analysis. PLoS One. 2022;17(1):e0263139. doi: 10.1371/journal.pone.0263139 35085366 PMC8794132

[4] Nicholas C. The orang asli and the contest for resources: Indigenous politics, development and identity in Peninsular Malaysia. Copenhagen: International Work Group for Indigenous Affairs; Center for Orang Asli Concerns. 2000.

[5] Adenan AZ, Mohd AAS, Mohd Feizal NH, Yaacob SS, Ibrahim K. Non-Communicable Diseases Among Orang Asli in Malaysia: A Scoping Review of Prevalence and Associated Factors. MJSSH. 2025;10(1):e003207. doi: 10.47405/mjssh.v10i1.3207

[6] Mahmud MH, Baharudin UM, Md Isa Z. Diseases among Orang Asli community in Malaysia: a systematic review. BMC Public Health. 2022;22(1):2090. doi: 10.1186/s12889-022-14449-2 36384509 PMC9670659

[7] Law LS, Sulaiman N, Gan WY, Adznam SN, Mohd Taib MN. Predictors of Overweight and Obesity and Its Consequences among Senoi Orang Asli (Indigenous People) Women in Perak, Malaysia. Int J Environ Res Public Health. 2020;17(7):2354. doi: 10.3390/ijerph17072354 32244318 PMC7178050

[8] Tay JEF, Ulaganathan V, Kua GYL, Adan MA, Lim SY. Nutritional Status of Orang Asli in Malaysia. Malays J Med Sci. 2022;29(3):17–29. doi: 10.21315/mjms2022.29.3.3 35846489 PMC9249419

[9] Chua EY, Mohd Shariff Z, Sulaiman N, Appannah G. Overweight and obesity among Orang Asli adults in Krau Wildlife Reserve, Pahang: A four-year follow-up study. Malays J Nutr. 2019;25(2):199–207.

[10] Tuan Abdul Aziz TA, Teh LK, Md Idris MH, Bannur Z, Ashari LS, Ismail AI, et al. Increased risks of cardiovascular diseases and insulin resistance among the Orang Asli in Peninsular Malaysia. BMC Public Health. 2016;16:284. doi: 10.1186/s12889-016-2848-9 27009064 PMC4806488

[11] Institute for Public Health(IPH). Technical Report: Orang Asli Health Survey (OAHS) 2022. Ministry of Health Malaysia. 2024.

[12] Osman A, Tan TT, Sakinah O, Khalid BA, Wu LL, Ng ML. Prevalence of NIDDM and impaired glucose tolerance in Aborigines and Malays in Malaysia. Diabetes Care. 1993;16(1):68–75. doi: 10.2337/diacare.16.1.68 8422835

[13] Mohamud WN, Suraiami M. Prevalence of diabetes, impaired fasting glucose and metabolic syndrome among female orang asli community in peninsular Malaysia. Int J Diab Dev Ctries. 2010;30(3):118. doi: 10.4103/0973-3930.66503

[14] Wong CY, Zalilah MS, Chua EY, Norhasmah S, Chin YS, Siti Nur’Asyura A. Double-burden of malnutrition among the indigenous peoples (Orang Asli) of Peninsular Malaysia. BMC Public Health. 2015;15:680. doi: 10.1186/s12889-015-2058-x 26194643 PMC4508822

[15] Whitehead SJ, Ford C, Gama R. A combined laboratory and field evaluation of the Cholestech LDX and CardioChek PA point-of-care testing lipid and glucose analysers. Ann Clin Biochem. 2014;51(Pt 1):54–67. doi: 10.1177/0004563213482890 23880620

[16] Definition and diagnosis of diabetes mellitus and intermediate hyperglycaemia: report of a WHO/IDF consultation. Geneva: World Health Organization. 2006. http://www.who.int/iris/handle/10665/43588

[17] Obesity: preventing and managing the global epidemic. 2000. 11234459

[18] Ithnin M, Juliana M, Mohamad Nor N ‘Ayn U, Mohd Effendy N, Mohd Rani MD. Knowledge, attitude, and practices of non-communicable diseases: comparison between orang asli and malay from rural area in negeri sembilan, malaysia: a comparative study. MJPHM. 2020;20(2):131–40. doi: 10.37268/mjphm/vol.20/no.2/art.411

[19] Chaturvedi N, Voight BF, Wells JC, Pritlove C. Race, ethnicity and ancestry in global diabetes research: grappling with complexity to advance equity and scientific integrity - a narrative review and viewpoint. Diabetologia. 2025;68(11):2318–26. doi: 10.1007/s00125-025-06516-1 40770495 PMC12534298

[20] Pacheco LS, Hernández-Ontiveros DA, Iniguez-Stevens E, Brodine S, Garfein RS, Santibañez M, et al. Prevalence and correlates of diabetes and metabolic syndrome in a rural indigenous community in Baja California, Mexico. BMC Public Health. 2018;18(1):1397. doi: 10.1186/s12889-018-6276-x 30572860 PMC6302508

[21] Mohamed SF, Mwangi M, Mutua MK, Kibachio J, Hussein A, Ndegwa Z, et al. Prevalence and factors associated with pre-diabetes and diabetes mellitus in Kenya: results from a national survey. BMC Public Health. 2018;18(Suppl 3):1215. doi: 10.1186/s12889-018-6053-x 30400865 PMC6218998

[22] Aronson R, Edelman SV, Burton G, Grover SA. Cardiovascular disease and diabetes: A scientific statement from the American Heart Association and the American Diabetes Association. Circulation. 2020;141(25):e900–30. doi: 10.1161/CIR.0000000000000764

[23] Miyakawa M, Shimizu T, Van Dat N. Prevalence, perception and factors associated with diabetes mellitus among adults in central Vietnam. BMC Public Health. 2017;17:298. 28381223 10.1186/s12889-017-4208-9 PMC5382364

[24] Dereje N, Earsido A, Temam L, Abebe A. Prevalence and Associated Factors of Diabetes Mellitus in Hosanna Town, Southern Ethiopia. Ann Glob Health. 2020;86(1):18. doi: 10.5334/aogh.2663 32140428 PMC7047764

[25] Li Y, Jiang Y, Lin J, Wang D, Wang C, Wang F. Prevalence and associated factors of diabetes mellitus among adults in Xiaoshan District, China. BMJ Open. 2022;12(3):e049754. doi: 10.1136/bmjopen-2021-049754 PMC8928303 35296469

[26] Aynalem SB, Zeleke AJ. Prevalence of Diabetes Mellitus and Its Risk Factors among Individuals Aged 15 Years and Above in Mizan-Aman Town, Southwest Ethiopia, 2016: A Cross Sectional Study. Int J Endocrinol. 2018;2018:9317987. doi: 10.1155/2018/9317987 29853887 PMC5944196

[27] Urrutia I, Martín-Nieto A, Martínez R, Casanovas-Marsal JO, Aguayo A, Del Olmo J, et al. Incidence of diabetes mellitus and associated risk factors in the adult population of the Basque country, Spain. Sci Rep. 2021;11(1):3016. doi: 10.1038/s41598-021-82548-y 33542348 PMC7862431

[28] Flor LS, Campos MR. The prevalence of diabetes mellitus and its associated factors in the Brazilian adult population. Rev Bras Epidemiol. 2017;20(1):16–29. doi: 10.1590/1980-5497201700010002 28513791

[29] Malta DC, Bernal RTI, se Sá ACMGN, da Silva TMR, Iser BPM, Duncan BB, et al. Self-reported diabetes and factors associated with it in the Brazilian adult population: National Health Survey, 2019. Cien Saude Colet. 2022;27(7):2643–53. doi: 10.1590/1413-81232022277.02572022 35730835

[30] Basit A, Fawwad A, Qureshi H, Shera AS. Prevalence of diabetes, pre-diabetes and associated risk factors: Second National Diabetes Survey of Pakistan (NDSP), 2016–2017. BMJ Open. 2018;8(6):e020961. doi: 10.1136/bmjopen-2017-020961 PMC6078264 30082350

[31] López-Jaramillo P, Sánchez RA, Diaz M, Cobos L, Bryce A, Parra-Carrillo JZ, et al. Latin American consensus on hypertension in patients with diabetes type 2 and metabolic syndrome. Arq Bras Endocrinol Metabol. 2014;58(3):205–25. doi: 10.1590/0004-2730000003019 24863082

[32] Einarson TR, Acs A, Ludwig C, Panton UH. Prevalence of cardiovascular disease in type 2 diabetes: A systematic literature review. Cardiovasc Diabetol. 2018;17:83. doi: 10.1186/s12933-018-0728-6 29884191 PMC5994068

[33] Einarson TR, Acs A, Ludwig C, Panton UH. Prevalence of cardiovascular disease in type 2 diabetes: a systematic literature review of scientific evidence from across the world in 2007-2017. Cardiovasc Diabetol. 2018;17(1):83. doi: 10.1186/s12933-018-0728-6 29884191 PMC5994068

[34] American Diabetes Association Professional Practice Committee. 10. Cardiovascular Disease and Risk Management: Standards of Care in Diabetes-2024. Diabetes Care. 2024;47(Suppl 1):S179–218. doi: 10.2337/dc24-S010 38078592 PMC10725811

